# Caprylic and Polygalacturonic Acid Combinations for Eradication of Microbial Organisms Embedded in Biofilm

**DOI:** 10.3389/fmicb.2017.01999

**Published:** 2017-10-18

**Authors:** Joel Rosenblatt, Ruth A. Reitzel, Nylev Vargas-Cruz, Anne-Marie Chaftari, Ray Hachem, Issam Raad

**Affiliations:** Department of Infectious Diseases, Infection Control & Employee Health, University of Texas MD Anderson Cancer Center, Houston, TX, United States

**Keywords:** caprylic acid, polygalacturonic acid, synergy, biofilm eradication, infection

## Abstract

There is a need for non-antibiotic, antimicrobial compositions with low toxicity capable of broad-spectrum eradication of pathogenic biofilms in food preparation and healthcare settings. In this study we demonstrated complete biofilm eradication within 60 min with synergistic combinations of caprylic and polygalacturonic (PG) acids in an *in vitro* biofilm eradication model against representative hospital and foodborne infectious pathogen biofilms (methicillin-resistant *Staphylococcus aureus*, multidrug-resistant *Pseudomonas aeruginosa*, *Candida albicans*, *Escherichia coli*, and *Salmonella enteritidis*). Antimicrobial synergy against biofilms was demonstrated by quantifying viable organisms remaining in biofilms exposed to caprylic acid alone, PG acid alone, or combinations of the two. The combinations also synergistically inhibited growth of planktonic organisms. Toxicity of the combination was assessed *in vitro* on L929 fibroblasts incubated with extracts of caprylic and PG acid combinations using the Alamar Blue metabolic activity assay and the Trypan Blue exclusion cell viability assay. The extracts did not produce cytotoxic responses relative to untreated control fibroblasts.

## Introduction

Biofilms are three-dimensional dense communities of microbial cells attached to surfaces that are encased in protective biopolymer matrices secreted by the embedded microbes (Costerton et al., 1999; Donlan and Costerton, 2002). Novel disinfecting approaches to rapidly eradicate microbial biofilms that are broad spectrum, economical, and that do not encourage development of resistant organisms remain a significant need in applications including prevention of illnesses from food preparation and storage as well as prevention and treatment of infections in healthcare settings conducive to biofilm formation such as on dermal wounds and implanted medical devices. The Centers for Disease Control and Prevention report that thousands of people get sick annually from consuming contaminated foods resulting in substantial health and economic burdens (National Center for Emerging and Zoonotic Infectious Diseases, and Division of Foodborne, Waterborne, and Environmental Diseases, 2016). Biofilms formed on surfaces of food processing and storage equipment can be very recalcitrant to eradication and can lead to food spoilage and other related economic costs (Van Houdt and Michiels, 2010). Similarly, a significant proportion of human microbial infections have been reported to be associated with biofilms (Miquel et al., 2016). Consequently, effective disinfection technologies that eradicate biofilms are needed for prevention and treatment of biofilm-based infections. Infections where biofilms are present can be especially challenging to treat. For example, biofilms can impair healing of wounds in microbially colonized environments, such as the colon, rectum, and urinary tract (Donlan and Costerton, 2002). Biofilms are also frequently present in chronic dermal wounds and contribute to their recalcitrance in healing (James et al., 2008). Furthermore, surgical site infections in the presence of implanted medical devices pose significant healthcare challenges whose complications are magnified when microbial biofilms form on the device surfaces (Magill et al., 2014). *Candida* species are the most prevalent fungal colonizer of human tissues and produce a significant proportion of medical device-associated fungemias (Nobile and Johnson, 2015).

A variety of approaches for eradicating biofilms in food handling and healthcare settings have been studied. These include synthetic and naturally derived antimicrobial agents as well as energy-based disinfection (Miquel et al., 2016). Traditional disinfectants are harsh chemical agents that can present undesirable mammalian and ecological toxicities (Christen et al., 2017). Traditional antiseptic agents have also demonstrated mammalian toxicities (Kim et al., 2017). Antibiotics, which are frequently naturally derived have been attractive solutions in these settings because of their lower toxicities. However, the wide use of antibiotics has led to development of antibiotic-resistant (including multi-drug resistant) organisms (Centers for Disease Control and Prevention, 2013). Biofilms resist penetration of antibiotics in part due to their extracellular matrix polysaccharides which can bind or otherwise restrict diffusion of antibiotics. Cells in the biofilm phenotype can also exhibit altered metabolic and other behaviors rendering them less susceptible to antibiotics (Van Acker et al., 2014). Additionally, the increasing prevalence of antibiotic-resistant biofilms (Centers for Disease Control and Prevention, 2013) impairs the efficacy of antibiotic therapies. Fungal biofilms can be similarly resistant to traditional antifungal therapeutics due to filtration and binding by their extracellular matrices, and can further reduce antifungal susceptibility through efflux pumps and other biofilm phenotype-specific mechanisms that protect them from antifungal-induced oxidative stresses (Van Acker et al., 2014). Due to the adaptation and continued evolution of increased resistance of biofilms to antibiotics and antifungals, there remains a significant need for improved non-antibiotic compositions that can effectively eradicate microbial biofilms without eliciting antibiotic resistance. Agents that are highly active against a narrow spectrum of organisms can select for biofilm formation by organisms they have weaker activity against. Optimal biofilm disinfection technologies therefore would have low cost, present minimal digestive, topical, and parenteral toxicities and would be capable of rapidly eradicating a wide spectrum of pathogenic microbes including Gram-positive, Gram-negative, and fungal pathogens. Plant-derived natural agents have been researched for their ability to provide optimal biofilm disinfection without undesirable accompanying safety or resistance effects.

Caprylic acid (CAP) is a medium chain fatty acid naturally present in coconut oil and mammalian (including human) breast milk (Jensen et al., 1990; Marina et al., 2009). CAP has been a component of some intravenously administered total parenteral nutrition formulations (Wanten and Calder, 2007; Rayyan et al., 2012). Antimicrobial effects have been reported for protonated CAP – most likely due to its ability to interact with the lipophilic portions of microbial cell membranes and disrupt cell membrane integrity (Pohl et al., 2011). The pK of CAP has been reported as approximately 4.8 (CRC, 2004–2005). In the protonated state, since CAP is a small, uncharged lipophilic molecule, its properties promote rapid penetration of biofilm and intercalation into embedded microbial cell membranes.

Polygalacturonic (PG) acid is naturally derived from pectin which is a structural biopolymer (polysaccharide) present in vegetable and fruit cell walls. Naturally derived PG is partially esterified (usually methoxylated), is usually derived from citrus rind or apple pomace, and may contain minor components of other sugar molecules in its backbone (Sriamornsak, 2013). The pK of PG varies with degree of esterification but ranges from about 3.5 to 4.1. At pHs above the pK, PG is usually soluble; however, at pHs below its pK it can form gels (Sriamornsak, 2013). The molecular weight of naturally derived PG is typically between 50 and 150 KDa (Sriamornsak, 2013). PG is widely used as a pharmaceutical excipient, in hydrocolloid wound dressings (Munarin et al., 2012) and has been shown to promote antimicrobial activity (Espitia et al., 2014). In light of these properties and since PG has also been shown to be effective in emulsifying lipids (Akhtar et al., 2002) here we studied the biocompatibility and potential synergy of the combination of PG and CAP for biofilm eradication in food handling and medical applications.

## Materials and Methods

### Microbial Strains

Testing was conducted using highly virulent Gram-positive, Gram-negative, and yeast pathogens representative of common hospital-acquired (HAI) and food-borne infections. Pathogens tested were methicillin-resistant *Staphylococcus aureus* (MRSA, MDA 120), multi-drug resistant (MDR) *Pseudomonas aeruginosa* (MDA 118), *Candida albicans* (MDA 117), *Escherichia coli* (MDA 123), and *Salmonella enteritidis* (ATCC 13076). All MDA numbered pathogens tested were clinical isolates cultured from blood of cancer patients with bacteremia stored in the MD Anderson Infectious Disease organism bank. The *S. enteritidis* strain was an ATCC standard. For testing organisms were grown from glycerol stock trypticase soy agar + 5% sheep blood (for bacteria) or sabouraud dextrose agar (for yeast). Each organism was inoculated into Muller Hinton Broth (MHB) and diluted to 0.5 McFarland. Further dilutions were made as necessary for testing.

### Inhibition of Planktonic Organisms

To assess potential antimicrobial activity of PG and CAP against planktonic organisms, MIC (PG or CAP) and Checkerboard (PG + CAP) assays were conducted. MICs were determined by microbroth dilutions in accordance with Clinical and Laboratory Standards Institute (CLSI) M07 guidelines (CLSI, 2015). Using round bottom 96-well plates, both HAI and food pathogens were exposed to a range of dilutions of PG (0, 0.25, 0.5, 0.75, and 1%), independently and in combinations (checkerboard) with a range of dilutions of CAP (0, 0.01, 0.02, 0.03, 0.04, and 0.05%). Sets of experiments were run where the plates were shaken and not shaken during incubation. MIC was determined by visual scoring for growth. The well with the lowest concentration of drug in which no turbidity was observed corresponded with the MIC for the organism tested.

### Time to Biofilm Eradication Testing

A well-established biofilm colonization model (Kuhn et al., 2002) was employed to test eradication of pathogenic biofilms following different durations of exposure to antimicrobial agents. Briefly, 1 cm silicone disks were placed in 24-well flat bottom cell culture plates and exposed to 1 ml of human plasma overnight at 37°C. Biofilm was established on silicone disks by inoculating with challenge organism (1 ml of 5.5 × 10^5^ CFU/ml) and incubating shaking at 37°C for 24 h. All culture liquid was then removed and disks were washed shaking for 30 min in isotonic sterile saline to remove any remaining planktonic organisms. After washing, disks were exposed at 37°C for 30 or 60 min independently to 1 ml of each test. After exposure, any biofilm remaining on the surface of the silicone disks was assessed by disrupting biofilm via sonicating disks in 5 ml isotonic saline for 15 min. The resulting solution was serially diluted and quantitatively cultured onto Trypticase Soy Agar + 5% sheep blood (bacteria) or Sabouraud Dextrose Agar (yeast). Complete eradication of the biofilm requires a recovery of no viable colonies following treatment. Recovery of fewer viable organisms than from the control indicates partial eradication of the biofilm. Each time point for each organism for each disinfecting solution was tested with six replicates. To ensure eradication was complete (no surviving dormant or persister cells) from biofilms for which no viable colonies were recovered following the exposure to disinfecting solutions, regrowth experiments were conducted by first exposing biofilm-colonized disks to each experimental solution, then rinsing and subsequently transferring the disks to fresh broth and re-incubating for an additional 24 h. Following the 24 h regrowth interval, disks were then sonicated and cultured as indicated above to determine whether any organisms remaining embedded in the biofilm were still viable.

Antimicrobial solutions were prepared containing 1% PG (Sigma-Aldrich, St. Louis, MO, United States, <15% esterified) + either 0.1 or 0.4% CAP (Sigma-Aldrich, St. Louis, MO, United States). 0.1% CAP was selected because it was near the reported aqueous solubility limit for CAP (Hoerr et al., 1946). The emulsifying capacity of pectins for lipids has been reported near 40% or more (Liang et al., 2015), hence 1% PG and 0.4% CAP (40% of the PG concentration) were selected for testing since 0.4% CAP was well above the solubility limit of CAP and therefore presented the potential for increasing the effective concentration of CAP presented to microbes. MHB, 1% PG, 0.1% CAP, and 0.4% CAP were included as control solutions. For consistency, the pH of all test solutions, including MBH control, was maintained at pH 4.25 with sodium hydroxide or hydrochloric acid as necessary.

### Mammalian Cytotoxicity Testing

Mammalian cytotoxicity testing was conducting following the indirect method of de Gomes et al. (2011) where L929 mouse fibroblasts are exposed to extracts of challenge compositions over a concentration range from 2 to 0.5% for 24 h (de Gomes et al., 2011). After exposure, viability was tested using the Alamar Blue Assay (O’Brien et al., 2000) and the Trypan Blue Exclusion staining assay (Strober, 2015). L929 fibroblasts (ATCC #CCL-1; ATCC, Manassas, VA, United States) were grown in Dulbecco’s modified Eagle’s medium (DMEM; Corning Cell Grow, Manassas, VA, United States) supplemented with 10% heat-inactivated fetal bovine serum (FBS; Sigma-Aldrich, St. Louis, MO, United States) in 5% CO_2_ (v/v) at 37°C. Seeding density of fibroblasts was 4.5 × 10^3^ cells/well in 96-well flat bottomed culture plates for Alamar Blue assay and 2.8 × 10^5^ cells in 25 cm^2^ culture flasks for live dead staining. Following the indirect exposure method of de Gomes et al. (2011), when growth reached approximately 60% confluence cells were exposed to a 2, 1, and 0.5% extracts of 1% PG + 0.4% CAP in DMEM + 10% FBS for 24 h. Control (untreated) cells were incubated in DMEM + 10% FBS without added disinfecting agents. After the exposures, cell viability and toxicity were assessed. All experiments were performed in triplicate.

The Alamar Blue assay (Life Technologies, Corp., Carlsbad, CA, United States) was used to assess the sensitivity of metabolic activity of fibroblasts following exposure to PG + CAP. This assay measures the overall metabolic activity based on production of highly fluorescent resorufin by reducing resazurin in response to enzyme activity in cells (O’Brien et al., 2000). Cytotoxic compounds cause fibroblasts to lose their ability to metabolically reduce resazurin to resorufin thus do not produce the fluorescent signal. After 24 h exposure to PG + CAP solutions, medium was replaced with 100 μl of Hank’s Balanced Salt Solution (HBSS; Corning Cell Grow, Manassas, VA, United States) + 10% Alamar Blue reagent and incubated for 4 h in 5% CO_2_ (v/v) at 37°C. Absorbance was determined at 570 nm using a microplate reader spectrophotometer. Cell viability (absorbance) was compared between treated and untreated control cells. Control (untreated) cells were incubated in DMEM + 10% FBS and their metabolic activity was assessed by the Alamar Blue assay described above. All experiments were performed in triplicate. Results are expressed as a percentage of fluorescent signal normalized to untreated controls.

The Trypan Blue exclusion test was used as a cell staining test to determine the number of viable cells present in cell suspension (Strober, 2015). Live cells with intact membranes have the ability to exclude the dye Trypan Blue, whereas dead cells do not. Therefore, viable cells had a clear cytoplasm whereas dead cells were stained with blue cytoplasm (Strober, 2015). After 24 h exposure to PG + CAP, cells were washed with HBSS to remove any anti-trypsin serum proteins and harvested from the culture flask with 0.05% trypsin EDTA (Corning Cell Grow, Manassas, VA, United States). Once detached, DMEM + 10% FBS was added and cells were pelleted at 200 × *g* for 7 min. Supernatant was decanted and cells were resuspended in 2 ml of HBSS. Ten microliter of aliquots of cell suspension was stained with 10 μl 0.4% Trypan Blue (Sigma-Aldrich, St. Louis, MO, United States). Live and dead cells were counted with a hemacytometer. Control, untreated cells were incubated in DMEM + 10% FBS and viability was assessed using the Trypan Blue exclusion test described above. All experiments were performed in triplicate. Results were expressed as percent viable cells in suspension.

### Statistical Testing

The Kruskal–Wallis test was used to determine any significant differences in the medians in any of the solutions tested. Subsequently pairwise comparisons were conducted using the Mann–Whitney *U*-test to compare performance of 1% PG + 0.4% CAP, individual components, and MHB control at the either 30 or 60 min time points. All comparisons were two-sided with an alpha level of 0.5. A *p-*value less than 0.05 (*p* < 0.05) was considered to be statistically significant.

## Results

### Inhibition of Planktonic Organisms

All MICs for individual components of CAP and PG as well as combinations are presented in **Table 1**. No differences were seen when the plates were shaken or not shaken. For MRSA, inhibition occurred at PG concentrations above 0.5% (its MIC). Growth of MRSA was completely inhibited at 0.03% CAP (its MIC). At 0.01% CAP, 0.25% PG in combination was able to inhibit MRSA growth. For *P. aeruginosa*, inhibition occurred at PG concentrations above 0.75%. Concentrations of CAP at 0.05% or less did not inhibit *P. aeruginosa*. At 0.05% CAP, a PG concentration of 0.25% in combination was able to inhibit *P. aeruginosa* growth. *CA* was not inhibited at PG concentrations of 1% or less nor at CAP concentrations of 0.05% or less. Nevertheless, at 0.01% CAP a PG concentration of 0.25% in combination was able to inhibit growth of *C. ablicans*. For *E. coli*, the MIC of PG was 1%. Concentrations of 0.05% or less CAP did not inhibit growth of *E. coli*. At a CAP concentration of 0.02%, 0.75% PG in combination was able to inhibit growth of *E. coli* and similarly at a CAP concentration of 0.04%, 0.5% PG in combination was able to inhibit growth of *E. coli*. *S. enteritidis* was not inhibited by PG at concentrations of 1% or less nor was its growth inhibited at CAP concentrations of 0.05% or less. At 0.01% CAP, 0.75% PG in combination was able to inhibit growth of *S. enteritidis* and similarly at 0.02% CAP, 0.5% PG in combination was able to inhibit growth of *S. enteritidis*.

**Table 1:** Minimum inhibitory concentration (MIC) of caprylic acid, polygalacturonic acid, and combinations.

	MIC PG alone	MIC CAP alone	MIC PG + CAP
MRSA	0.5%	0.03%	0.25/0.01%
*Ps. aerguinosa*	0.75%	>0.05%	0.25/0.05%
*C. albicans*	>1%	>0.05%	0.25/0.01%
*E. coli*	1%	>0.05%	0.75/0.02%
			0.5/0.04%
*S. enteridits*	>1%	>0.05%	0.75/0.01%
			0.5/0.02%


*MICs were determined by microbroth dilutions in accordance with CSLI (Clinical and Laboratory Standards Institute) M07 guidelines. Pathogens were exposed to a range of dilutions of PG (0, 0.25, 0.5, 0.75, and 1%), independently and in combinations (checkerboard) with a range of dilutions of CAP (0, 0.01, 0.02, 0.03, 0.04, and 0.05%). MIC was determined by visual scoring for growth (“>” denotes greater than)*.

### Time to Biofilm Eradication

**FIGURE 1:**
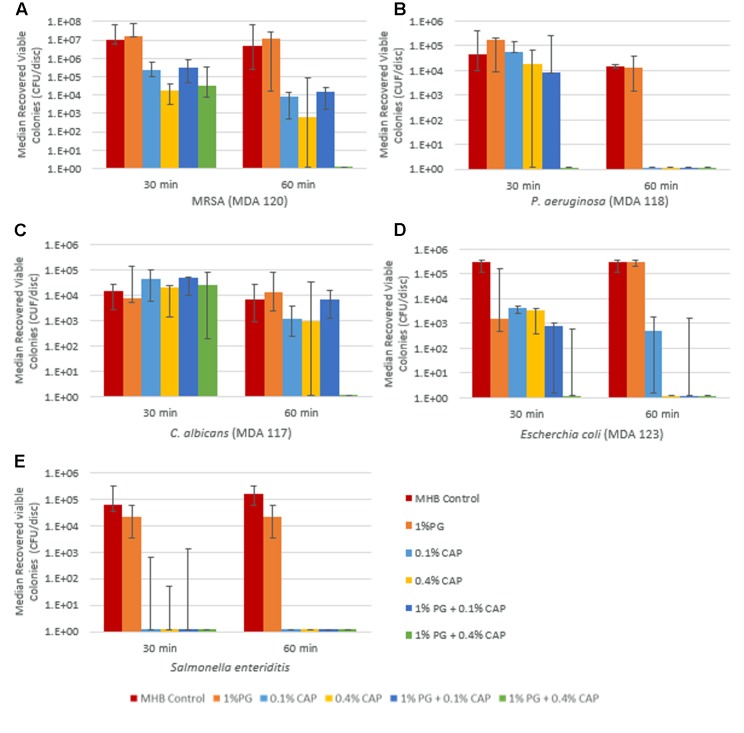
Synergistic eradication of biofilms from representative infectious pathogens – MRSA **(A)**, *P. aeruginosa* **(B)**, *C. albicans* **(C)**, *E. coli* **(D)**, and *S. enteritidis* **(E)** by polygalacturonic acid (PG) and caprylic acid (CAP) combinations. All figures are presented as median recovered viable colonies with range bars.

Time to biofilm eradication testing results for broth control, 1% PG, 0.1% CAP, 0.4% CAP, and for the combinations 1% PG + 0.1% CAP and 1% PG + 0.4% CAP are presented in **Figures 1A–E** for MRSA, *P. aeruginosa*, *C. albicans*, *E. coli*, and *S. enteritidis*, respectively, section “Results” presented are the median colony forming units per silicone disk with range bars for the six replicates. Efficacy of 1% PG + 0.4% CAP in eradicating biofilm was statistically significant for MRSA (60 min), *P. aeruginosa* (30 min), and *C. albicans* (60 min) relative to MHB control (*p* = 0.002 for all organisms) and 1% PG alone (*p* = 0.002, *p* = 0.015, and *p* = 0.002, respectively). Compared to 0.4% CAP alone, 1% PG + 0.4% CAP displayed significant reductions against *P. aeruginosa* at 30 min (*p* = 0.041) and *C. albicans* at 60 min (*p* = 0.015). For MRSA 1% PG + 0.4% CAP completely eradicated biofilm (no viable colonies recovered) while 0.4% CAP alone had a median growth of 6.25 × 10^2^ CFU per disk, however, this difference did not attain statistical significance (*p* = 0.18). One percent of PG + 0.4% CAP completely eradicated *E. coli* within 30 min (no viable colonies recovered) while 0.4% CAP had a median growth of 3.55 × 10^3^ CFU per disk; however, this difference also did not attain statistical significance (*p* = 0.2). Both CAP alone and the combination of PG + CAP was highly effective in eradication *S. enteritidis* biofilm within 30 min of exposure. There was no regrowth for any solution for which no viable colonies were recovered in the biofilm eradication assay. This verified that eradication was indeed complete when no viable colonies were recovered in the biofilm eradication assay.

### Cytotoxicity Testing

**FIGURE 2:**
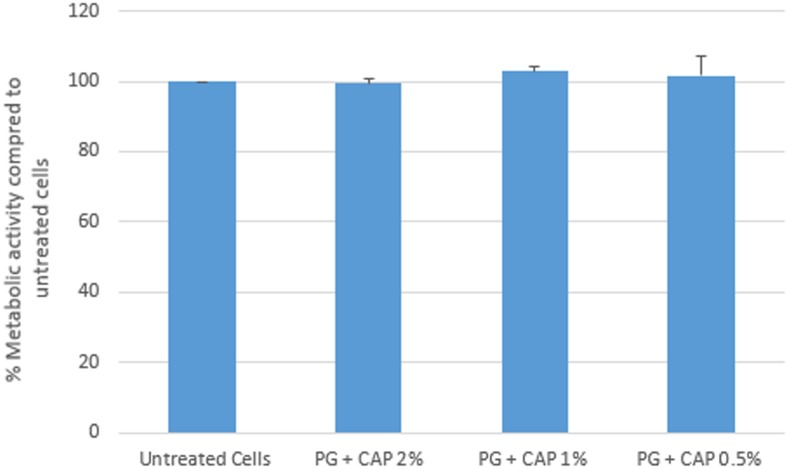
*In vitro* cytotoxicity by Alamar Blue metabolic activity assay. L-929 fibroblasts were treated with PG + CAP extracts for 24 h. Cell metabolic activity was assessed with the Alamar Blue assay. Results are expressed as median percentage metabolic activity relative to untreated control cells with range bars.

**Figure 2** presents results of the Alamar Blue cytotoxicity assay for control fibroblasts as well as fibroblasts exposed to 2, 1, and 0.5% extracts of 1% PG + 0.4% CAP. Results are expressed as percentage fluorescence signal relative to the control. There were no significant differences (*p* > 0.31) in metabolic activity between any of the groups compared to untreated control cells. Based on counting 1.5–2.0 × 10^6^ cells in each of three replicates, the control in the Trypan Blue cell exclusion assay yielded an average 96.9% viable cells (clear cells). The 2% extract of 1% PG + 0.4% CAP yielded an average 97.6% viable cells (**Table 2**). There was no significant difference in cell viability between the groups (*p* = 0.41).

**Table 2:** *In vitro* cytotoxicity assessment of cell viability by Trypan Blue staining: L-929 Fibroblasts were treated with PG + CAP extracts for 24 h.

	Untreated L929 Cells (cells/mL)	L929 Fibroblasts treated with 1% PG + 0.4% CAP(cells/mL)
Mean Live Cells ± standard deviation	1.84 ± 0.18 × 10^6^	1.57 ± 0.11 × 10^6^
Mean Dead Cells ± standard deviation	6.00 ± 2.12 × 10^4^	3.83 ± 0.60 × 10^4^
% Viable	96.89%	97.62%


*Cell viability was assessed with the Trypan Blue exclusion assay. Results are expressed as percentage viable cells relative to untreated control cells*.

## Discussion

As shown in **Figure 1**, protonated CAP at both 0.1 and 0.4% concentrations demonstrated reduction in viable MRSA and *C. albicans* organisms in biofilms after 60 min incubation, however, complete eradication was only seen against *P. aeruginosa* biofilms within 60 min. Previously, CAP was reported to have antimicrobial activity against planktonic Gram-positive and Gram-negative organisms including *P. aeruginosa* and MRSA (Kim and Rhee, 2016) but its effectiveness against biofilm was not assessed. Other than for *E. coli* (**Figure 2**), there was no significant impact of increasing the CAP concentration from 0.1 to 0.4% on biofilm eradication when no PG was present. This is consistent with the reported aqueous solubility limit of CAP of approximately 0.07% (Hoerr et al., 1946), hence once the CAP concentration approached saturation there was little impact on biofilm eradication from having insoluble CAP present. Although PG alone demonstrated inhibitory activity against planktonic *P. aeruginosa*, MRSA, and *E. coli* there was no appreciable antimicrobial effect against *P. aeruginosa*, *E. coli*, and MRSA biofilms. This may have been a result of the molecular size and electrostatic charge of PG whose access to embedded microbial cells within biofilms may have been reduced by interactions with extracellular biofilm matrix polysaccharides. PG alone had limited effectiveness against planktonic *C. albicans* and *S. enteritidis* or biofilms of *C. albicans* and *S. enteritidis*.

In this study, a synergistic reduction in the time to biofilm eradication was seen with the 1% PG + 0.4% CAP combination against MRSA (at 60 min), *P. aeruginosa* (at 30 min), *E. coli* (at 30 min), and *C. albicans* (at 60 min). One percent PG + 0.1% CAP demonstrated synergistic reduction in time to biofilm eradication against *E. coli* (60 min). The PG + CAP combinations were also highly effective against *S. enteritidis* biofilms, but CAP alone was also highly effective against *S. enteritidis* biofilms at the exposures tested (30 and 60 min). Since the broth for the controls was adjusted to the same pH (4.25) as the antimicrobial solutions, the antimicrobial effects were not due to solution acidity. It is possible that synergy of the PG + CAP combinations against *S. enteritidis* biofilms was present at exposure times shorter than those tested in this study. A hypothesis for the enhanced synergy seen with 0.4% CAP in combination with PG may be that the apparent solubility of CAP is enhanced in the PG + CAP combination and the bioavailability of both CAP and PG within biofilms is significantly increased when combined. At 0.4% concentration, CAP alone (without PG) showed visible signs of phase separation or immiscibility and was well above the reported aqueous CAP solubility limit of 0.07–0.08% at 25°C (Hoerr et al., 1946). This is also consistent with the minor differences in antimicrobial activity seen between CAP alone at 0.1 and 0.4% in **Figure 1** where soluble CAP might have saturated. Observations of 1% PG + 0.4% CAP through an optical microscope showed the presence of finely emulsified oil droplets consistent with prior reports of the lipid emulsifying properties of PG (Akhtar et al., 2002). The planktonic data for PG and CAP combinations support an additional hypothesis for dual mechanisms of antimicrobial synergy for the combination beyond increasing the bioavailability of CAP and PG within the biofilms. While protonated CAP is known to directly disrupt cell membranes (Pohl et al., 2011), PG is capable of binding or precipitating important metal ions and molecules with cationic residues (Espitia et al., 2014) such as proteins and peptides. These activities inhibit microbial proliferation and survival, particularly when cell membranes are simultaneously stressed and made more porous by the action of protonated CAP.

Caprylic acid is a naturally occurring fatty acid. In the ionized (deprotonated) state (neutral pH), caprylate ion is a nutrient with a well-established metabolic profile in mammals (Hirabara et al., 2006). CAP can become ionized when the pH rises above 4.8 (CRC, 2004–2005), hence in most physiological environments CAP will rapidly ionize to a benign nutrient. In a topical formulation, protonated CAP which retains antimicrobial activity can be present by maintaining a pH below 4.8. This is within the natural pH range seen in human skin (Lambers et al., 2006). Additional epithelial environments such as the vaginal canal (Lang, 1955) and digestive tract (Evans et al., 1988) also naturally maintain acidic pHs. Dermal wounds have been reported to exhibit improved healing at acidic pHs (Schreml et al., 2010). Honey, which has a pH of 3.2–4.2 (Pieper, 2009) has been directly applied to and used to treat wounds. Some clinical trials have reported an enhancement of healing with topical application of honey and the safe topical use of acidic, medical grade honey in wounds has been consistently reported (Lee et al., 2011). Wound healing has also been reported to be aided by the presence of CAP in wound beds (Pieper and Caliri, 2003; Srivastava and Durgaprasad, 2008). Due to its pK, PG naturally maintains a pH in the same range as honey. PG has been widely used in hydrocolloid wound dressings with reported benefits of maintaining a moist, acidic environment, and providing a bacterial barrier and is in several commercial wound healing sheet and paste products (Munarin et al., 2012).

## Conclusion

We found that PG and CAP combinations are capable of rapidly eradicating representative Gram-positive, Gram-negative, and fungal biofilms within 60 min. We further found that the combination does not induce a cytotoxic response in mammalian fibroblasts. This in addition to the prior history of safe use of the individual components in wound healing and nutrition applications suggests that the combination should be suitable for topical and food disinfection uses. The synergistic, non-antibiotic, antimicrobial combination requires further *in vivo* testing to substantiate both its safety and efficacy.

## Author Contributions

JR, RR, and IR designed the study. RR and NV-C performed planktonic, biofilm, and cytotoxicity assays. JR, RR, NV-C, A-MC, RH, and IR reviewed and analyzed data and contributed conclusions. JR and RR wrote the manuscript. NV-C, A-MC, RH, and IR edited the manuscript. All authors read, commented on, and approved the final manuscript.

## Conflict of Interest Statement

The authors declare that the research was conducted in the absence of any commercial or financial relationships that could be construed as a potential conflict of interest.
